# Effects of Physical Activity and Mental Health on Chewing Difficulty in South Korean Adults: A Cross-Sectional Analysis

**DOI:** 10.3390/healthcare13162004

**Published:** 2025-08-14

**Authors:** Mi-Ra Lee

**Affiliations:** Department of Dental Hygiene, Hanseo University, Seosan 31962, Republic of Korea; leemra1@hanmail.net; Tel.: +82-41-660-1576

**Keywords:** adults, chewing difficulty, mental health, oral health, physical activity

## Abstract

Objectives: This study aimed to investigate the effects of physical activity and mental health on chewing difficulty in South Korean adults using data from the seventh Korea National Health and Nutrition Examination Survey (KNHANES VII, 2016–2018). Methods: A total of 8212 participants aged ≥20 years who completed surveys on their physical activity, mental health, and chewing status were included. Chewing difficulty was assessed via a five-point Likert scale based on self-reported discomfort when chewing due to oral problems, and dichotomized for analysis. The participants were divided into two groups: those aged <40 and those aged ≥40 years. Complex sample analyses, including cross-tabulations and logistic regression, were used to examine the associations. Results: Among men aged <40, high perceived stress was associated with 1.71 times higher odds of experiencing chewing difficulty (*p* < 0.05). For men aged ≥40, high perceived stress (OR of 1.45) and a sleep duration of <7 h (OR of 1.32) were significant risk factors (*p* < 0.05). In women aged ≥40 years, high Patient Health Questionnaire 9 (PHQ-9) scores (≥10; OR of 2.35) and high perceived stress (OR of 1.64) were significantly associated with chewing difficulty (*p* < 0.001). Conclusions: Perceived stress, insufficient sleep, and depression are significant mental health factors associated with chewing difficulty in adults. These findings emphasize the necessity of a multifaceted approach, including psychosocial mental health issues, to effectively manage chewing difficulty in this population.

## 1. Introduction

An individual’s chewing ability not only encompasses the mechanical breakdown of food to facilitate swallowing and digestion, but also plays a pivotal role in influencing their nutritional intake. This function is integral to maintaining an individual’s overall health and quality of life [1]. Furthermore, chewing has been demonstrated to alleviate stress and contribute to the regulation of cognitive functions, including attention and executive functioning [2]. Accordingly, the preservation and enhancement of chewing abilities are critical components of healthy aging [3]. A decline in chewing abilities is widely recognized as a common functional limitation among older adults [4], yet it may also manifest in younger populations as a consequence of prolonged deterioration in their oral health. Specifically, reductions in chewing capacity are frequently observed in individuals suffering from dental caries, periodontal disease, or tooth loss [5]. Moreover, impaired chewing ability has been associated with a range of factors, including low socioeconomic status, restrictions in daily activities attributable to health conditions, depressive symptoms, smoking, and reduced utilization of dental care services [4]. According to a study by Rémond et al. [6], reduced masticatory efficiency negatively affects postprandial protein metabolism, potentially hindering the maintenance of muscle mass. This suggests that masticatory problems may impair protein intake and utilization, ultimately leading to muscle loss and decreased physical activity.

Regular physical activity plays a critical role in both the primary and secondary prevention of various chronic diseases and is associated with a reduced risk of premature mortality [7], offering substantial benefits for both physical and mental health [8]. Conversely, physical inactivity is a recognized risk factor for a range of chronic conditions, including cardiovascular disease, cancer, diabetes, obesity, hypertension, musculoskeletal disorders, and depression [7]. According to the World Health Organization (WHO) [8], as of 2022, 31% of adults and 80% of adolescents failed to meet the recommended levels of physical activity. Moreover, aging is associated with a decline in both the quantity and quality of skeletal muscle, including musculature in the oral and maxillofacial regions. The strength of the cheeks and lips is essential for effective oral bolus control and suppression. During mastication and swallowing, proper muscle tone in these regions prevents the intrusion of food into the oral vestibule between the gums and cheeks. In contrast, diminished oral muscular strength may result in discomfort during chewing, leading individuals to preferentially consume softer foods. This in turn may suppress the activation of the masticatory muscles and perpetuate a cycle of further oral muscle weakening [9].

Mental health problems are among the most prevalent medical conditions globally, and individuals with such conditions are at an increased risk of physical illnesses, including diabetes, cardiovascular disease, and cancer [10]. People with mental health issues are also more susceptible to dental and oral health problems due to factors such as poor self-care, the side effects of medications, and dental anxiety [11]. In particular, depression can affect both physical and functional aspects of oral health through various mechanisms, serving as a potential risk factor for oral health issues such as difficulty in chewing [12]. In a population-based retrospective cohort study, Liao et al. [13] reported that individuals with depression had an elevated risk of developing temporomandibular disorders (TMD). Similarly, a prospective cohort study by Kindler et al. [14] identified depression as a risk factor for TMD-related pain, which may negatively affect chewing abilities. Furthermore, a bidirectional relationship has been observed between oral health and depression [15]. A study on older adults found that the prevalence of depression was nearly twice as high in those with chewing difficulties compared to those without [16]. A systematic review also reported an increased risk of depression in individuals who experienced difficulty with chewing or speaking [15]. Additionally, individuals’ oral health status has been associated not only with depression, but also with other mental and psychological conditions, such as anxiety and stress [12].

Previous studies have demonstrated that subjective and objective measurements of masticatory function do not always correspond, as these aspects of chewing abilities may be influenced by distinct factors; therefore, comprehensive evaluation and management of chewing function should consider both physical and psychological components [17]. Although prior studies in both domestic and international contexts have explored factors related to chewing abilities—including physical activity [18], cognitive function [2], depression [4,16,17], individuals’ nutritional status and quality of life [3], and oral diseases [1]—most have focused primarily on older adults. However, because chewing abilities influence dietary choices and nutritional intake and play a central role in an individual’s overall well-being and quality of life, it is necessary to extend the research scope to encompass the general adult population. Beginning at around age 40—a critical period of life transition—adults experience systemic and oral tissue aging, which increases the risk of oral health problems such as periodontal disease and contributes to a decline in chewing ability [4,19]. Therefore, it is important to examine masticatory problems in adults, particularly by distinguishing between those under and over the age of 40. Accordingly, the present study aimed to investigate the impact of physical activity and mental health on chewing difficulty among South Korean adults using data from the Korea National Health and Nutrition Examination Survey (KNHANES). The findings of this study are expected to serve as a foundational resource for future research and policy development in this field.

## 2. Materials and Methods

### 2.1. Participants

**Figure 1:**
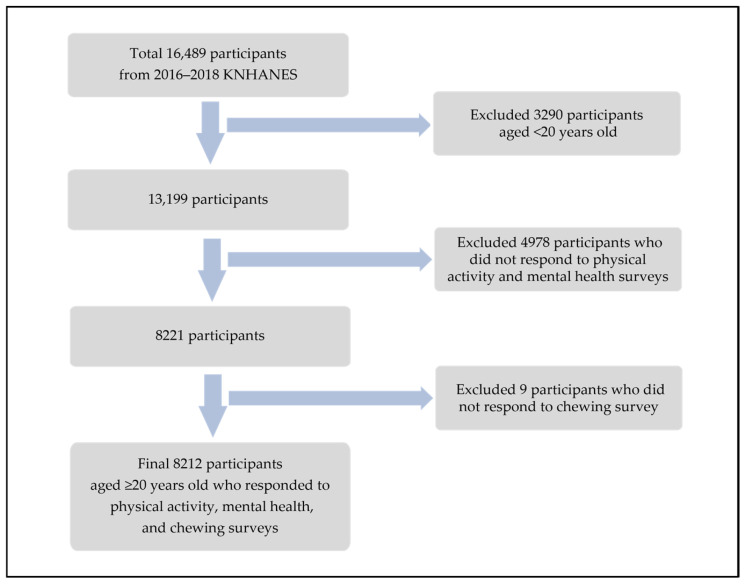
Flow chart for study participants selecting process.

This study utilized data from the seventh KNHANES, conducted between 2016 and 2018 by the Korea Disease Control and Prevention Agency (KDCA). The KNHANES employed the most recent census data available at the time of the sample design as the primary sampling frame. A stratified, multistage clustered probability sampling method was used, in which survey districts and households were selected in two stages. All individuals aged 1 year and older residing in the selected households were included as eligible participants, thereby ensuring the extraction of a nationally representative sample of the target population, namely South Korean residents [20]. From a total of 16,489 participants in the 2016–2018 KNHANES, 13,199 adults aged 20 years and older were initially selected. To ensure analytical consistency across all variables. Participants were excluded if they had missing data on any of the following variables: (1) physical activity (vigorous activity, moderate activity, walking, or strength training), (2) mental health (depressive symptoms measured by the Patient Health Questionnaire 9 (PHQ-9), perceived stress level, or average weekday sleep duration), or (3) chewing difficulty. After excluding incomplete cases, 8212 individuals with complete responses to all relevant variables were included in the final analysis (Figure 1).

The study protocol was approved by the Institutional Review Board of Hanseo University (IRB HS24-08-13).

### 2.2. Measurements

The dependent variable in this study was chewing difficulty, derived from the Health Behavior Survey component of the KNHANES. The participants were asked the following question: “Do you currently experience discomfort when chewing food due to problems with your teeth, dentures, gums, or other oral issues?” The responses were recorded on a five-point Likert scale: “very uncomfortable,” “uncomfortable,” “neutral,” “not uncomfortable,” and “not uncomfortable at all.” For analysis, we used a dichotomized variable provided by the KNHANES statistics team, which categorized “very uncomfortable” and “uncomfortable” as indicating chewing problems, and the remaining responses as indicating no chewing problems [20]. The independent variables included physical activity indicators obtained from the Health Behavior Survey, specifically the number of days of walking, strength training, and aerobic physical activity. The number of days spent walking and performing strength training per week was categorized into three groups: “none,” “1–2 days per week,” and “3 or more days per week.” Aerobic physical activity was assessed using a dichotomous variable provided by the KNHANES statistics team. Participants were classified as meeting the aerobic activity recommendation if they engaged in at least 150 min of moderate-intensity physical activity, 75 min of vigorous-intensity physical activity, or an equivalent combination per week (with 1 min of vigorous activity counted as 2 min of moderate activity) [20]. Mental health variables were also derived from the Health Behavior Survey and included depressive symptoms assessed using the PHQ-9, perceived stress levels, and the average sleep duration on weekdays. The PHQ-9 is a validated depression screening tool consisting of nine items with a total score ranging from 0 to 27. Scores of 10 or above are classified as indicating depression [21]. Perceived stress was assessed using a binary variable provided by the KNHANES statistics team: responses of “feeling very much” and “feeling much” were categorized as high stress, while “feeling a little” and “hardly feeling any” were categorized as low stress [20]. Average weekday sleep duration was recorded as a continuous variable and dichotomized based on the median value. The control variables included sociodemographic characteristics (household income and educational level) and oral health behaviors (frequency of daily tooth brushing, use of oral hygiene products, dental checkups within the last year, and smoking status). Additional covariates included oral health status variables (experiences of toothache in the past year, number of decayed permanent teeth, periodontal disease, and condition of dental prostheses) and general health conditions (hypertension and diabetes).

### 2.3. Statistical Analyses

As the KNHANES employed a two-stage stratified cluster sampling design, all the analyses in the present study accounted for this, including stratification, clustering, and sampling weights, to ensure that the findings could be generalized to the entire South Korean population. The participants were categorized into two age groups: those aged <40 years and those aged ≥40 years. To examine the associations between chewing difficulty and variables such as participants’ sociodemographic characteristics, oral health behaviors and status, general health conditions, physical activity, and mental health, complex sample cross-tabulations were performed. To identify the factors influencing chewing difficulty, complex sample multivariate logistic regression analyses were conducted. These models were adjusted for covariates, including participants’ sociodemographic characteristics, oral health behaviors and status, and general health status. In addition, multicollinearity among independent variables was assessed using the variance inflation factor (VIF), with values <10 indicating no multicollinearity issues. All the statistical analyses were performed using IBM SPSS Statistics version 20.0 (SPSS Inc., Chicago, IL, USA), and a *p*-value of <0.05 was considered statistically significant.

## 3. Results

### 3.1. Associations Between Participant’ Characteristics and Chewing Difficulty by Age Group

Table 1 shows the associations between participants’ characteristics and chewing difficulty according to the age group (<40 vs. ≥40). In both age groups, a higher prevalence of chewing difficulty was observed among men, individuals in the first and second household income quartiles, current smokers, those who had experienced a toothache within the last year, had periodontal disease or hypertension, or had partial or complete dentures compared to their respective counterparts. Additionally, among participants aged ≥40 years, chewing difficulty was more frequently reported by those who had a high school education or less, were widowed or divorced, brushed their teeth fewer than three times per day, did not use oral hygiene products, had not received a dental checkup in the last year, had seven or more decayed permanent teeth, or had diabetes compared to their respective counterparts. These associations were statistically significant (*p* < 0.05) (Table 1).

### 3.2. Associations Between Physical Activity, Mental Health, and Chewing Difficulty by Age Group

Table 2 shows the associations between physical activity, mental health, and chewing difficulty according to age group (<40 vs. ≥40 years). In both age groups, participants who walked on fewer days per week, did not engage in aerobic physical activity, or reported higher levels of perceived stress had a higher prevalence of chewing difficulty compared to those who did not. Additionally, among participants aged ≥40 years, a significantly higher prevalence of chewing difficulty was observed in individuals who did not participate in strength training and those with a PHQ-9 score of ≥10 (*p* < 0.05) (Table 2).

### 3.3. Factors Affecting Chewing Difficulty by Age Group

Table 3 presents the factors influencing chewing difficulty according to sex and the age group (<40 vs. ≥40 years). The explanatory power of the logistic regression models, based on Nagelkerke’s R^2^, was 0.156 for men and 0.138 for women <40 and 0.223 for men and 0.239 for women ≥40. VIF values for the independent variables—walking days, strength training days, aerobic physical activity, PHQ-9, perceived stress, and weekday sleep duration—ranged from 1.006 to 1.199, indicating an absence of multicollinearity among the predictors. After adjusting for covariates, high perceived stress was significantly associated with increased odds of chewing difficulty among both men aged <40 years (OR of 1.71) and those aged ≥40 years (OR of 1.45). Additionally, among men aged ≥40 years, sleeping <7 h on weekdays was associated with higher odds of chewing difficulty (OR of 1.32). In women aged ≥40 years, both depressive symptoms (PHQ-9 score ≥ 10; OR of 2.35) and high perceived stress (OR of 1.64) were significantly associated with chewing difficulty (*p* < 0.05) (Table 3).

## 4. Discussion

Oral well-being, encompassing the ability to chew and speak, plays a crucial role in social interactions and is an important component of quality of life [4]. In particular, chewing ability is vital for maintaining a healthy diet that supports physical health and cognitive function [22]. Therefore, this study aimed to examine whether physical activity and mental health affected chewing difficulty among South Korean adults, stratified by aged <40 years or ≥40 years. First, the cross-tabulation analysis (Table 2) showed that the frequency of walking and aerobic physical activity were potentially associated with chewing difficulty in both adults aged <40 years and those aged ≥40 years. Specifically, fewer days of walking and a lack of aerobic physical activity were associated with masticatory problems. Additionally, among those aged ≥40 years, fewer days of strength training were linked to chewing difficulty. This finding aligns with a study by Jang and Kim [18], who reported that individuals who performed strength exercises had a 0.105 lower prevalence of chewing difficulties compared to those who did not. However, in our multivariate logistic regression analysis, none of the physical activity factors showed a statistically significant association with chewing difficulty, which contrasts with their findings [18]. This discrepancy may suggest that the simple correlations identified in the cross-tabulations were attenuated in the multivariate model or that the effect of physical activity on chewing difficulty may have been moderated by other factors such as mental health, oral health status, or socioeconomic characteristics. Moreover, this may also reflect limitations in our use of frequency-based, self-reported physical activity measures, which did not account for intensity or duration. It is also important to consider that subjective chewing difficulty may not be directly linked to physical activity levels alone, but rather influenced by a complex interplay of psychological, physiological, and social factors. Future research should incorporate more detailed and validated metrics for physical activity—including intensity and duration—along with objective assessments of chewing function to more clearly elucidate these associations. Physical activity refers to any bodily movement produced by skeletal muscles that results in energy expenditure [8]. Routine physical activity reduces the incidence of chronic diseases and premature mortality and enhances both physical and psychological well-being [7]. Since oral health is linked to systemic health, including chronic diseases, engaging in regular and active physical activity may contribute to the maintenance of oral health.

Regarding the mental health factors related to chewing difficulty, perceived stress was significant in both age groups. The multivariate logistic regression analysis showed that individuals perceiving high stress levels had greater odds of developing chewing difficulty than those perceiving low stress levels: 1.71 times higher in men under 40, 1.45 times higher in men aged 40 and above, and 1.64 times higher in women aged 40 and above. This finding is consistent with Kim and Kwon’s study, which reported that groups with greater chewing difficulties experienced more stress than normal groups [23]. Stress is a physiological and psychological response to environmental changes and harmful stimuli, and stress-related physical and mental illnesses pose global health challenges [24]. Stress negatively influences oral health by affecting oral hygiene behaviors and habits essential for maintaining oral health [25]. Furthermore, saliva secreted during mastication protects the teeth and oral mucosa and is essential for both chewing and swallowing [26]. However, stress negatively affects salivary secretion and flow and immune functions [25], increases susceptibility to oral diseases, and exacerbates conditions such as periodontal disease and temporomandibular disorders [25]. In addition, depressive symptoms were associated with chewing difficulty in women aged ≥40 years. Those with a PHQ score of ≥10 had 2.35 times higher odds of having chewing difficulty than those scoring <10. This result aligns with the findings of Takagi et al. [17], Krause et al. [4], and Chun and Doo [16], who reported associations between depression and masticatory abilities. Depression manifests as a loss of interest or pleasure, sadness, guilt or low self-esteem, sleep or appetite disturbances, extreme fatigue, and impaired concentration [27]. Mental disorders such as depression correlate with individuals’ subjective oral health status [28]: individuals with depression tend to neglect their oral hygiene and are more likely to engage in behaviors detrimental to their oral health, such as smoking and alcohol consumption, thereby increasing their risk of various oral health problems [29]. Moreover, AlJameel et al. [12] reported that individuals who experienced depression in early adulthood were approximately twice as likely to experience chewing difficulties in old age as those without depression, and those with recurrent or prolonged depression faced even higher risks. Severe mental illness is also associated with a 3.4-fold higher probability of complete tooth loss and an increased incidence of dental caries compared to the general population [10]. Therefore, chewing difficulty should be recognized as a significant oral health concern, particularly among individuals with a severe mental illness. A particularly notable finding of this study is that a broader range of mental health factors—including weekday sleep duration, depression, and perceived stress—were more closely associated with chewing difficulty in adults aged ≥40 years compared to those aged <40. Middle-aged adults often face increased stress and loneliness due to family and social responsibilities, which can negatively affect their mental health [30]. Krause et al. [4] reported that one in five adults aged ≥55 years experiences reduced masticatory ability, emphasizing the importance of maintaining or improving chewing function to support healthy aging. These findings suggest the need for increased societal attention and preventive approaches targeting mental and oral health issues in midlife and beyond. Chewing difficulty may adversely impact both the quantity and quality of dietary intake, leading to a deterioration in overall quality of life and contributing to declines in both physical and mental health status [23]. Conversely, adequate masticatory function has been reported to exert beneficial effects on attention, cognitive performance, mood regulation, and stress alleviation [31]. In this context, the present study’s findings—demonstrating significant associations between chewing difficulty and mental health factors such as depressive symptoms, perceived stress, and insufficient sleep duration—underscore the potential contribution of mental health status as a critical determinant of masticatory function. These results highlight the need to conceptualize oral health not solely as a physiological domain, but as a multidimensional construct intricately linked to psychological and social well-being. Given these findings, the development and implementation of an integrated, interdisciplinary approach that addresses both mental and oral health is warranted. In particular, the establishment of early screening systems for individuals at elevated risk of mental health disorders, coupled with streamlined referral pathways to appropriate services, should be prioritized. For example, community health centers could incorporate combined oral and mental health screening tools into routine checkups and develop collaborative care pathways involving dentists, mental health professionals, and primary care providers. Early-intervention programs specifically targeting middle-aged adults—who may be at greater risk of both masticatory and psychological decline—could also be piloted and evaluated. Moreover, regular oral health assessments and tailored behavioral interventions targeting individuals vulnerable to mental health issues merit further investigation through longitudinal research to evaluate their potential role in preserving or improving masticatory function.

This study is not without limitations. First, due to the cross-sectional nature of the design, causal inferences cannot be established. Furthermore, the interaction effects among key mental health variables—such as perceived stress, depressive symptoms, and sleep duration—were not examined. Future studies should explore these interrelationships and their combined influence on chewing difficulty. In addition, the complex mediating or moderating pathways through which health-related factors—such as physical activity, dietary behaviors, and oral hygiene practices—affect chewing ability should be examined through longitudinal or intervention-based research to elucidate causal mechanisms more precisely. Second, the analysis did not account for several potential confounders, including dietary intake patterns, use of medications, and comorbid conditions, which may have influenced the observed associations. Third, chewing difficulty was assessed through self-reported responses, with those indicating “very uncomfortable” and “uncomfortable” classified as experiencing masticatory problems. While this dichotomization was intended to reflect a clinically meaningful level of functional impairment, it may oversimplify the continuum of chewing function. Future studies should incorporate validated assessment tools and objective measures to enhance the reliability and accuracy of chewing difficulty classification. Nonetheless, subjective evaluation is meaningful, as the discomfort experienced while chewing is a critical factor. This study’s significance lies in its identification of the factors influencing chewing difficulty among South Korean adults, stratified by age as being <40 or ≥40 years, and the attention it calls to mental health variables such as stress as important contributors to chewing difficulty.

## 5. Conclusions

This study aimed to investigate the impact of physical activity and mental health on chewing difficulty in South Korean adults using data from the seventh KNHANES (2016–2018), with a focus on individuals around 40 years of age. In men aged <40 years, high perceived stress was associated with 1.71 times higher odds of chewing difficulty. In men aged ≥40 years, high perceived stress (OR of 1.45) and sleeping for <7 h (OR of 1.32) increased the likelihood of chewing difficulty. In women aged ≥40 years, those with a PHQ-9 score of ≥10 (OR of 2.35) and high perceived stress (OR of 1.64) were more likely to experience chewing difficulty. Perceived stress, inadequate sleep, and depressive symptoms were identified as significant mental health factors associated with chewing difficulty among adults. These findings underscore the necessity of adopting a comprehensive and multidisciplinary approach that addresses the psychological and social dimensions of mental health in order to effectively manage chewing difficulty in the adult population.

## Figures and Tables

**Table 1:** Associations between participants’ characteristics and chewing difficulty by age group (<40 vs. ≥40 years).

Characteristics	Category	<40 Years			≥40 Years		
		No Chewing Difficulty	Chewing Difficulty	*p* *	No Chewing Difficulty	Chewing Difficulty	*p* *
Sociodemographic characteristics							
Sex	Men	992 (91.3)	100 (8.7)	0.047	1751 (69.5)	736 (30.5)	0.002
	Women	1197 (93.6)	92 (6.4)		2411 (73.6)	933 (26.4)	
Household income	1st and 2nd quartiles	681 (89.1)	82 (10.9)	<0.001	1704 (62.9)	1052 (37.1)	<0.001
	3rd and 4th quartiles	1507 (94.1)	110 (5.9)		2446 (79.8)	615 (20.2)	
Education level	≤High school	879 (91.7)	82 (8.3)	0.244	2748 (67.1)	1411 (32.9)	<0.001
	≥College	1310 (93.1)	110 (6.9)		1414 (84.4)	254 (15.6)	
Marital status	Married	1055 (92.4)	97 (7.6)	0.251	3366 (74.4)	1182 (25.6)	<0.001
	Single	1109 (92.9)	91 (7.1)		143 (71.0)	57 (29.0)	
	Widowed or divorced	25 (83.3)	4 (16.7)		652 (61.8)	430 (38.2)	
Oral health behaviors							
Tooth-brushing frequency	<3 times	892 (92.0)	87 (8.0)	0.491	1983 (66.7)	1026 (33.3)	<0.001
	≥3 times	1297 (92.9)	105 (7.1)		2179 (77.4)	643 (22.6)	
Use of oral hygiene devices	Yes	1331 (92.9)	111 (7.1)	0.461	2292 (77.3)	681 (22.7)	<0.001
	No	858 (92.0)	81 (8.0)		1870 (66.4)	988 (33.6)	
Dental examination within 1 year	Yes	785(93.0)	66 (7.0)	0.593	1645 (79.1)	457 (20.9)	<0.001
	No	1404 (92.3)	126 (7.7)		2517 (67.9)	1212 (32.1)	
Smoking	Yes	474 (87.5)	70 (12.5)	<0.001	607 (63.4)	331 (36.6)	<0.001
	No	1715 (94.0)	122 (6.0)		3555 (73.5)	1338 (26.5)	
Oral health status							
Toothache within 1 year	Yes	487 (85.3)	90 (14.7)	<0.001	1091 (56.9)	808 (43.1)	<0.001
	No	1702 (94.8)	102 (5.2)		3068 (78.8)	861 (21.1)	
DMFT ^†^	<7	1041 (93.7)	76 (6.3)	0.073	1952 (75.0)	664 (25.0)	<0.001
	≥7	1148 (91.5)	116 (8.5)		2210 (69.5)	1005 (30.5)	
Periodontal disease	Yes	195 (84.4)	32 (15.6)	<0.001	1388 (64.1)	769 (35.9)	<0.001
	No	1994 (93.4)	159 (6.6)		2637 (78.5)	764 (21.5)	
Prosthesis status	None	1960 (93.0)	162 (7.0)	0.040	2182 (80.2)	559 (19.8)	<0.001
	Fixed prosthesis	224 (89.3)	28 (10.7)		1522 (72.6)	586 (27.4)	
	Partial or complete dentures	5 (74.3)	2 (25.7)		458 (47.8)	524 (52.2)	
General health conditions							
Hypertension	Normal	1565 (93.2)	124 (6.8)	0.025	1502 (77.8)	433 (22.2)	<0.001
	Prehypertension	462 (92.0)	44 (8.0)		1070 (74.9)	366 (25.1)	
	Hypertension	159 (87.3)	24 (12.7)		1586 (65.6)	868 (34.4)	
Diabetes	Normal	1783 (93.1)	146 (6.9)	0.277	2246 (75.8)	725 (24,2)	<0.001
	Impaired fasting	259 (90.3)	31 (9.7)		1125 (71.1)	472 (28.9)	
	Diabetes	37 (93.4)	3 (0.6)		577 (63.7)	347 (36.3)	

* Based on complex sample chi-squared test. ^†^ Separated by median value. Abbreviations: DMFT, decayed, missing, or filled permanent teeth.

**Table 2:** Associations between physical activity, mental health, and chewing difficulty by age group (<40 vs. ≥40 years).

Characteristics	Category	<40 Years			≥40 years		
		No Chewing Difficulty	Chewing Difficulty	*p* *	No Chewing Difficulty	Chewing Difficulty	*p* *
Physical activity							
Days of walking per week	None	272 (88.6)	40 (11.4)	0.013	782 (63.9)	462 (36.1)	<0.001
	1–2 days	380 (91.1)	33 (8.9)		661 (69.6)	296 (30.4)	
	≥3 days	1537 (93.6)	119 (6.4)		2719 (75.1)	911 (24.9)	
Days of strength training per week	None	1576 (92.0)	150 (8.0)	0.270	3154 (70.1)	1397 (29.9)	<0.001
	1–2 days	248 (94.5)	17 (5.5)		303 (74.8)	84 (25.2)	
	≥3 days	365 (93.6)	25 (6.4)		705 (79.8)	188 (20.0)	
Compliance with aerobic activity recommendations	Non-compliant	1004 (91.1)	106 (8.9)	0.021	2415 (68.8)	1125 (31.2)	<0.001
	Compliant	1185 (93.7)	86 (6.3)		1747 (76.5)	544 (23.5)	
Mental health							
PHQ-9 score (2016, 2018)	<10	2074 (92.7)	176 (7.3)	0.127	4026 (73.3)	1498 (26.7)	<0.001
	≥10	115 (89.0)	16 (11.0)		136 (45.9)	171 (54.1)	
Perceived stress level	Low	1442 (93.7)	108 (6.3)	0.003	3338 (74.3)	1163 (25.7)	<0.001
	High	747 (90.3)	84 (9.7)		824 (63.7)	506 (36.3)	
Average weekday sleep duration ^†^	<7 h	731 (91.7)	70 (8.3)	0.288	1635 (72.0)	658 (28.0)	0.944
	≥7 h	1458 (93.0)	122 (7.0)		2525 (71.9)	1008 (28.1)	

* Based on complex sample chi-squared test. ^†^ Separated by median value.

**Table 3:** Factors affecting chewing difficulty by sex and age group (<40 vs. ≥40 years).

Variables	Category	<40 Years		≥40 Years	
		Men OR (95% CI)	Women OR (95% CI)	Men OR (95% CI)	Women OR (95% CI)
Physical activity					
Days of walking per week	None	1.16 (0.58–2.35)	2.05 (0.92–4.54)	1.10 (0.78–1.56)	1.11 (0.85–1.44)
	1–2 days	0.86 (0.40–1.86)	1.70 (0.87–3.33)	1.17 (0.81–1.68)	1.16 (0.83–1.61)
	≥3 days	1.00	1.00	1.00	1.00
Days of strength training per week	None	0.98 (0.53–1.82)	1.46 (0.51–4.19)	1.04 (0.76–1.44)	1.16 (0.81–1.66)
	1–2 days	0.82 (0.35–1.93)	0.37 (0.06–2.49)	1.55 (0.99–2.43)	1.22 (0.65–2.27)
	≥3 days	1.00	1.00	1.00	1.00
Compliance with aerobic activity recommendations	Non-compliant	1.42 (0.84–2.39)	0.92 (0.49–1.70)	1.04 (0.81–1.34)	1.02 (0.80–1.32)
	Compliant	1.00	1.00	1.00	1.00
Mental health					
PHQ-9 score (2016, 2018)	<10	0.53 (0.14–1.95)	1.77 (0.83–3.79)	1.25 (0.74–2.12)	2.35 (1.50–3.69) **
	≥10	1.00	1.00	1.00	1.00
Perceived stress level	Low	1.71 (1.05–2.77) *	1.01 (0.61–1.68)	1.45 (1.09–1.93) *	1.64 (1.26–2.14) **
	High	1.00	1.00	1.00	1.00
Average weekday sleep duration ^†^	<7 h	0.91 (0.55–1.52)	1.41 (0.85–2.33)	1.32 (1.04–1.67) *	0.93 (0.75–1.16)
	≥7 h	1.00	1.00	1.00	1.00
Model fit (Nagelkerke’s R^2^)		0.156	0.138	0.223	0.239

OR, odds ratio; CI, confidence interval. Adjusted for confounding variables, including participants’ sociodemographic characteristics (household income, education level, marital status), oral health behaviors and status (tooth-brushing frequency, use of oral hygiene devices, dental examination within 1 year, smoking, toothache within 1 year, DMFT (decayed, missing, or filled permanent teeth), periodontal disease, prosthesis status), general health conditions (hypertension, diabetes, obesity), physical activity, and mental health. * *p* < 0.05, ** *p* < 0.001. ^†^ Separated by median value.

## Data Availability

The National Health and Nutrition Survey data disclose raw data on the Korea Centers for Disease Control and Prevention’s website, so consent to the collection and use of personal information and data was downloaded and used after user information registration (URL: https://knhanes.kdca.go.kr/knhanes/main.do#; accessed on 5 January 2025).

## Abbreviations

The following abbreviations are used in this manuscript:

WHO	World Health Organization
TMD	temporomandibular disorder
KNHANES	Korea National Health and Nutrition Examination Survey
KDCA	Korea Disease Control and Prevention Agency
PHQ-9	Patient Health Questionnaire 9
VIF	variance inflation factor
DMFT	decayed, missing, or filled teeth
